# Clinical Profile of COVID-19 in Children and Research Progress on Angiotensin-converting Enzyme 2: A Mini-review

**DOI:** 10.31729/jnma.5436

**Published:** 2020-10-31

**Authors:** Qian Gao, JingLiu, Zhilong Mu, Xianpeng Yan, Jay Narayan Shah, Fuyong Jiao

**Affiliations:** 1 Xi'an Medical University, Xi'an, China; 2 Children's Hospital of Shaanxi Provincial People's Hospital, 3rd Affiliated Hospital of Medical College of Xi'an Jiaotong University, Xi'an Shanxi, China; 3 Patan hospital, Patan Academy of Health Sciences, Lalitpur, Kathmandu, Nepal

**Keywords:** *children*, *Coronavirus Disease 2019 (COVID-19)*, *SARS-Cov-2*, *Angiotensin-Converting Enzyme 2 (ACE2)*

## Abstract

The cases of coronavirus disease 2019 in children have been increasing with the ongoing pandemic. The finding suggests children have mild symptoms and a short course of the disease. Angiotensinconverting enzyme-2 mediates entry of the virus into the cell, the combination of virus and ACE2 leads to an increase in activity of angiotensin II, resulting in acute injury to lungs, myocardium and other organs. The infection causes down-regulation of ACE2 expression. The ACE2 plays an important role in the infection progression and clinical characteristics of COVID-19. Works on ACE2 and virus spike protein have future prospects of strategic information on prevention, management as well as vaccine development.

## INTRODUCTION

In December 2019 cases of pneumonia of unknown cause reported from Wuhan, China was later found to be caused by Severe Acute Respiratory Syndrome Coronavirus 2 (SARS-CoV-2). Subsequently the disease casue by this novel corona virus pneumonia was named the coronavirus disease 2019 (COVID-19) by the World Health Organization.^1^ This virus is a coronavirus, singlestrand positive-sense RNA virus which bears 80% similar genome sequence to that of earlier welknown SARS corona virus.^2–4^ An outbreak of unusual pneumonia was reported in Wuhan with many cases linked to Huanan Seafood Market that sells seafood as well as live exotic animals. Two patients who developed acute respiratory syndromes after independent contact history with this market shared common clinical features including fever, cough, and multiple ground-glass opacities in the bilateral lung field with patchy infiltration. The COVID-19 quckly spread and was declared a pandemic of significant public health concern which has affected all 213 countries and territories around the world, with reported total cases of 17,745,673 and deaths reaching 682,197 with maximum numbers of infectgions and death in USA, as of July 31, 2020 Worldometer's update.^5^ In USA, New York City reported the infection fatality rate (IFR) of 1.4%.^6^ Further subgroup analysis of the data from New York revealed that children aged 0-17 years accounted for only 0.06%, and there was an exponential rise with increasing age.^7^ In this mini review we present and discuss the findings of extensive literature search for COVID-19 in children.

## METHODS

We performed an extensive review of advances in COVID-19 and the role of ACE2 in research progress. Search methodology included data repositories of ‘PubMed, Google Scholar, Google, Web of Science’ from the beginning of 2020 till June 2020. The following keywords, alone or in combination, were used: 'children, SARS-CoV-2, coronavirus disease 2019, COVID-19, angiotensinconverting enzyme 2, ACE2'. Titles and abstracts were assessed for relevance based on the contents of the full text. Articles for COVID-19 in children for its clinical presentation and role of ACE2 in infection, disease progression, and management were reviewed. The information was assessed and summarized on 'how children have a mild presentation of COVID-19, SARS-COV-2 infectivity, and future prospects of ACE2'.

## RESULTS

In this mini review, we found that infection by SARS-CoV-2 is less common in children, and has mild symptoms. The explanation for this presentation of COVID-19 in children is dueto immature immune system responses and insufficient expression of ACE2 protein. Less exposure of children due to early closer to schools due to lockdown has is thought to be a contributing factor for less spread in children. The physiological factors, for example, better regeneration capacity of alveolar epithelium, healthy endothelium in blood vessels in children, as well as absence of increased co-morbidities of cardiovascular, renal and respiratory system due to aging seen in adults play important role in infection and disease progression of COVID-19. The findings of the literature review are discussed in separate subheadings, focusing on clinical presentation, role of ACE2, and future prospects in research progress.

## DISCUSSION

### Children are less affected by COVID-19 and have mild symptoms

Children are less affected by COVID-19, accounting for less than 2%, and present with diverse but mild symptoms of fever, cough, runny nose, gastrointestinal symptoms, or no symptoms. Infection in children do not show significant gender difference. Recovery is quick, mostly within one week without servere complications possibly because children have healty endothelium in their blood vessels are less prone to inflammation and clotting causing damage to vital organs.^8–19^ Up to 25% of children in family clusters with exposure to positive adults were asymptomatic, an important aspect of transmission. Up to 25% of children in family clusters with exposure to positive adults do not show symptoms which is an important aspect of transmission dynamics to plan the strategy for control and prevention. Recovered children with negative nasopharyngeal reverse transcription polymerase chain reaction (RT-PCR) have shown continued positive anal swabs. Thus, swab tests from both ends are recommended and justified to declare them fully recovered, and also to avoid oral-fecal transmission.^20^

The mild presentation in children is related to specific features of novel corona virus and prsenece of ACE2 in children. The SARS-CoV-2 receptor binding domain (RBD) sS-protein have strong affinity with human ACE2, a property similar to other cororna virus (SARS COV) resembling to bat coronavirus HKU9-1.^21^ However, insufficient expression of ACE2 protein in children, combined with immature immune function and low immune response induced by the virus, children exibit less infetivity and mild clinical symptoms.^22^ According to the pathological studies of COVID-19 and SARS, a sharp decrease in T lymphocytes leads to the breakdown of the immune system. The cellular immune system of children differs from that of adults atypical clinical manifestations or even covert infection. The frequent childhood vaccinations and repeated pathogens infections might be resulting in trained immunity of innate immune cells, immune fitness of adaptive immune cells or cross-protection of antibodies in children.

The specific hemoglobin structure play important role in newborns as up to 80% of fetal hemoglobin is composed of alpha and gamma chains. This plays an important protective role because SARS-CoV-2 virus proteins are known to break down the 1-chain of hemoglobin to form iron porphyrin, interfering heme synthesis and oxygenation.^16^ Cross-immunization with other viruses and frequent upper respiratory tract infections up to 8 to 12 per year in children below 6 years of age also play a protective role as effective T cells can clear the virus.^26,27^ Strong ‘innate immune response’ of children due to live-vaccines and frequent viral infections help in the control of infection at the site of entry, unlike in adults with dysfunctional over-active innate immune response and suppressed adaptive immunity.

Children have better regeneration capacity of the alveolar epithelium, and healthy blood vesels with healthy endothelium. Also, they have non-existent comorbidities of adults due to aging, smoking, obesity, diabetes and cardiovascular disease, all of which play protective role, and thus children exibit milder symptoms and faster recovery from lung and cardiovascular injuries of COVID-19.^24^

The overall low proportion of children affected by COVID-19 (and other coronavirus diseases like SARS and MERS), may also be due to reduced exposure of children from early closure of schools, strict lockdown, combined with quarantine and isolation of infected adults.^24^

### ACE2 plays important role in SARS-CoV-2 infection and disease progression

Main host cell receptor for SARS-CoV in human is ACE2 and a deciding factor for the entry of virus into the cell. COVID-19 is initiated through the inoculation of SARS CoV-2 in the respiratory tract mucosa and cell entry facilitated by the ACE2 receptor by a process involving the transmembrane serine protease receptor 2 (TMPRSS2).^16^ This is followed by viremia and replication in the lung, and possibly the gastrointestinal tract. The ACE2 receptor is the key point for SARS-CoV-2 (also for other corona virus, e.g.SARS-COV), and a critical link between immunity, inflammation, and cardiovascular disease. It is present abundantly in the lungs, and also in the cardiovascular system, gut, kidney, central nervous system, and adipose tissue. As a negative regulator of the renin-angiotensin-aldosterone system (RAAS) and facilitator of amino acid transport, ACE2 affects blood pressure, fluid and electrolyte balance, systemic vascular resistance and protects against lung, heart and gut failure. After binding by SARS-CoV-2, there is a loss of ACE2 function driven by endocytosis and activation of proteolytic cleavage and processing. The rhACE2 (recombinant human ACE2), angiotensin analogs (Ang 1-7), and MAS receptor agonists can enhance ACE2 action and are potential therapies for disease conditions associated with an activated renin-angiotensin system.^31,32^

ACE2 is a multifunctional glycoprotein metalloprotease with both enzymatic (catalytic) and non-enzymatic (non-catalytic) functions, a key element in the protective arm of the renin-angiotensin system (RAS).^33^ It exists in two forms: membrane binding and solubility. Membrane-bound protein is a transmembrane protein with an extracellular domain, which is the receptor of the SARS-CoV-2 spinous process protein. The soluble form of circulating ACE2 can be cut and secreted as the outer domain of the N-terminal.^34^ The ACE2 converts angiotensin I (Ang I) into angiotensin-(1-9), and degrades angiotensin II (Ang II) into angiotensin-(1-7). When angiotensin 1-9 binds to MAS receptors, it antagonizes the classical RAS system, thus playing the role of anti-inflammation and reducing organ damage.^35^

The ACE2 is the invasion target of SARS-CoV-2 having 10 to 20-fold higher affinity compared to other corona virus (eg. SARS). The surface S protein of virus can trigger infection after binding to ACE2. Infection causes production of many inflammatory factors, like interleukin-1 (IL-1), interferon-gamma (IFN-gamma), tumor necrosis factor (TNF), etc down-regulate the expression of ACE2.^36^ This down-regulation activates the renin-angiotensin system (RAS) and damage to the heart, lungs, intestines, and other organs. Therefore, the difference in the expression or function of ACE2 and RAS blockers can increase the level of ACE2, and influence the severity of the infection. Although angiotensin enzyme inhibitors (ACEIs) does not directly regulate ACE2, both ACEIs and angiotensin receptor antagonists (ARBs) can indirectly increase the expression of ACE2. Animal studies have shown that the expression of ACE2 in ACEIs/ARBs-treated mice is significantly increased.^37^ Diabetic patients treated with ACEIs show elevated circulating ACE2 levels.^38^ All these factors explain why COVID-19 is less infectivity in children, show mild symptoms and recover quickly.

The wider spread of coronavirus can be explained to its higher affinity to ACE2. Compared to other cororna virus (e.g. earlier SARS-CoV), the three-dimensional spike S protein on the surface of novel SARS-CoV-2 have been observed to exhibit 10 to 20 times higher affinity and easier cell entry of virus via S protein coupling to ACE2 on the surface of pulmonary epithelium.^39^ Children have less ACE2 compared to adults. The expression of ACE2 is down-regulated by an infection-causing accumulation of angiotensin II. This accumulation results in an inflammatory response, leading to acute lung injury, ARDS, myocardial injury, and some times gastrointestinal manifestations of nausea and vomiting because of the systematic distribution of ACE2 in multiple organs.^40^ The serum angiotensin II level in patients with COVID-19 pneumonia is significantly higher than in healthy individuals and is linearly correlated with viral load.^41^ The ACE2 gene lies on the X-chromosome, Xp22.2. ACE2 is less in the female kidney because of the effects of the ovary, and presence of estradiol (E2), and opens an area of therapeutic possibility to target ACE2.^42^ Balancing ACE2 may improve the outcome of COVID-19 in both sexes by reducing inflammation, thrombosis, and death.^43^ Thus research on ACE2 has significant value in overall management of COVID-19.

### Future research prospects of ACE2 in management of COVID-19

Aggressive research and as a result the progress has been seen in various aspects of management of COVID-19 due to the serious public health effects globally. Efforts to develop for drug discovery and therapies for current novel corona virus, unlike the earlier experience of SARS, is based on its wider impact world wide. This impact is likely to last for a long period, arousing the interests of government to meet the demand of people. At the same time pharmaceutical companies see it as an economically viable endeavor to invest in research and production of new drugs and vaccines for a large global market.

Animal studies have shown improved lung function after inhaling recombinant ACE2, and the production of protective antibodies against S protein which may play important role in the treatment of COVID-19.^44^ It is recommended that interferon and lopinavir / ritonavir should be tried early, or ribavirin can be added. Interferon-gamma and lopinavir / ritonavir are more commonly used in clinical practice.^45^ Due to unique physiological characteristics, less number of cases, and mild presentation of COVID-19 in children, drugs and vaccine trials lag behind adults, and yet to be carried out on full scale. This is also because children are considered a vulnerable population in clinical trials and experimental studies.

Among the strategies in the vaccines development for novel corona virus, mainly its targeted to the surface spike S protein. Various modalities to target either full-length of S protein, or its receptor-binding domain (RBD), or virus-like particles (VLP) or vector based vaccine developemnt. The ACE2 plays important role being the bidnding site of virus, and facilitates entry of virus in to the cell. Unlike in previous SARS virus, there is only one potential hydrophobic interaction between RBD of SARS-CoV-2 and ACE2 (L79, M82). The specific RBD (F486) and one cation-π interaction ACE2 (K353)/RBD (Y492) (N Dong =-2020 F1000) have been found to be of interest.^46^ The property of virus-ACE2 interaction has a significant role in virus infectivity, pathogenicity and isease progression that directs the drug and vaccine development research.^47,48^ The ACE2 specific research has the potential for prevention and control of the COVID pandemic.

## WAY FORWARD

Children have mild symptoms of COVID-19 and a short course of the disease possibly because to their immature immune function and insufficient expression of ACE2. Works on ACE2 and virus spike protein have good research prospects in prevention-management- and vaccine development for COVID-19.
